# Does access to healthy food vary according to socioeconomic status and to food store type? an ecologic study

**DOI:** 10.1186/s12889-019-6975-y

**Published:** 2019-06-18

**Authors:** B. V. L. Costa, M. C. Menezes, C. D. L. Oliveira, S. A. Mingoti, P. C. Jaime, W. T. Caiaffa, A. C. S. Lopes

**Affiliations:** 1 Department of Nutrition. Researcher of Group of Interventions in Nutrition, Universidade Federal de Minas Gerais, 190 Prof. Alfredo Balena Ave, Belo Horizonte, MG 30.130-100 Brazil; 2 Department of Medicine, Researcher of Group of Interventions in Nutrition, Universidade Federal de São João del-Rei, Diamantina, 35. 501-296, Diamantina, Brazil; 3 Department of Statistics, Universidade Federal de Minas Gerais, 33937-280, Belo Horizonte, Brazil; 4 Department of Nutrition, Universidade de São Paulo, São Paulo, 01246-904 Brazil; 5 Department of Preventive and Social Medicine, Universidade Federal de Minas Gerais, 30130-100, Belo Horizonte, Brazil

**Keywords:** Commercial sectors, Environment and public health, Socioeconomic factors, Urban health

## Abstract

**Background:**

The food environment can influence opportunities and barriers to food access. This study aimed to investigate whether access to healthy foods varies according to store types and the socioeconomic status of the users of the public health promotion program in Brazil, known as the Health Academy Program.

**Methods:**

A total of 18 Health Academy Program centers were selected via simple conglomerate sampling. Health Academy Program users living up to 1 km from the food stores were evaluated (*n* = 2831). Their socioeconomic status was investigated via face-to-face interviews. The food stores were audited through direct observation. Variables included the community nutrition environment (type and location) and consumer nutrition environment (healthy food store index, involving variables such as availability, variety, and advertising of healthy and unhealthy products). Multiple linear regression analysis was performed to examine the association between access to healthy foods, socioeconomic status, and food store type.

**Results:**

A total of 336 stores were investigated. The majority were specialty fruit and vegetable markets/stores or open-air food markets. Access to healthy food was only associated with the food store type. An increase of 1% in the availability of specialized fruits and vegetable markets or open-air food markets and supermarket raised healthy food store index values by 0.12 and 0.07, respectively.

**Conclusions:**

Public food supply policies aimed at improving the diet quality of the population and reducing inequality in access should prioritize the implementation of stores of better quality, such as specialty fruit and vegetable markets and open-air food markets.

## Background

The food environment can influence opportunities or barriers to accessing food [1, 2]. Supermarkets, specialized fruit and vegetable markets, and open-air markets are stores that offer healthier food, relative to local markets, fast-food restaurants, small groceries and convenience stores [3]. However, it is important to consider that this relationship (classification of food and store types) might not be so simplistic. To illustrate, the same food store type might have different quality and offer different products according to the neighborhood. The stores might vary with respect to the availability, variety, and price of the food sold, which supports the discussion in the literature that food access is different across areas of varied socioeconomic status (SES) [3–8].

Studies in high-income countries have shown that individuals with favorable SES have greater access to supermarkets, and report higher levels of fruit and vegetable (FV) consumption [3–9]. In contrast, those with low SES live in areas with a higher number of small local markets and convenience stores, which offer limited variety and sell products of low quality (physical nature or condition of food) at higher prices [3, 5, 7]. These areas typically house a limited number of supermarkets and a greater concentration of fast-food outlets, suggesting greater exposure to unhealthy food [8, 9]. However, some studies do not support this evidence [10, 11].

Food environment characteristics appear to influence access to healthy and unhealthy food, which in turn impacts food consumption [9]. Although there is some consensus on the environmental contribution to diet and health, evidence regarding the food environment in Latin American countries is still scarce, especially since the studies only approach the community nutrition environment [8]. The lack of evidence in developing countries can lead to inappropriate policy planning and public intervention actions [12]. Given this gap, in countries such as Brazil, it is important to evaluate a range of characteristics and to contribute to efforts to answer important questions about access to healthy food. In Brazil, we do not know if individuals with different SES have different access to healthy food or if the access to healthy food varies according to food store type. These relationships have been explored mainly in American studies; however, these geographical associations might diverge [13]. Thus, to accurately identify and compare the dynamics of the food environment in different contexts, studies must be conducted in different parts of the world.

Therefore, this study aims to investigate whether access to healthy foods varies according to stores types and the SES level among the Health Academy Program (HAP) members in a Brazilian metropolis.

## Method

### Study design and setting

This study was conducted in Belo Horizonte, Minas Gerais State, Brazil. The municipality is the sixth largest in the country, with an urban population of 2,502,557 [14]. This research is an ecological study about the food environment conducted between March 2013 and June 2014 in the context of the Health Academy Program (HAP), a public service part of the Brazilian health system [15]. The HAP is a health promotion strategy that works with the implementation of public spaces, known as poles, gifted with infrastructure, equipment, and qualified professionals, aiming to create healthy environments by offering opportunities for regular physical exercise classes, healthy eating, and community education action at no cost. This program represents one of the main coping strategies for chronic noncommunicable diseases of the Brazilian government [15]. Understanding the food environment in HAP will make it possible to reformulate and elaborate public food and nutrition policies that favor the construction of a healthy food environment.

### Study sample

We used two data sources: (1) individual-level data from HAP participants; and (2) data on the HAP food environment, including information about food stores.

Data on the HAP food environment were obtained, which included a representative sample of the HAP centers. These poles offer opportunities for regular physical exercise and education activities, according to the following criteria: priority operation in the morning hours; location in a high and middle vulnerability area, as they are the predominant characteristics of the service in the municipality; and no participation in intervention studies within the preceding two years. The exclusion criteria were: location in areas of low vulnerability to health (*n* = 6) and participation in intervention related to food and nutrition in previous studies (*n* = 2). Forty-two of the fifty HAP centers were eligible at the time of the study [16].

Of these 42 HAP centers, 18 (two centers for each region) were selected to participate in the study via simple conglomerate sampling stratified according to the nine administrative regions of the city. Two poles were selected in each region because it was later decided to perform a community intervention study [17]. The sample was representative of HAP centers with 95% confidence and less than 1.4% error, based on the estimated population [16].

**Fig. 1:**
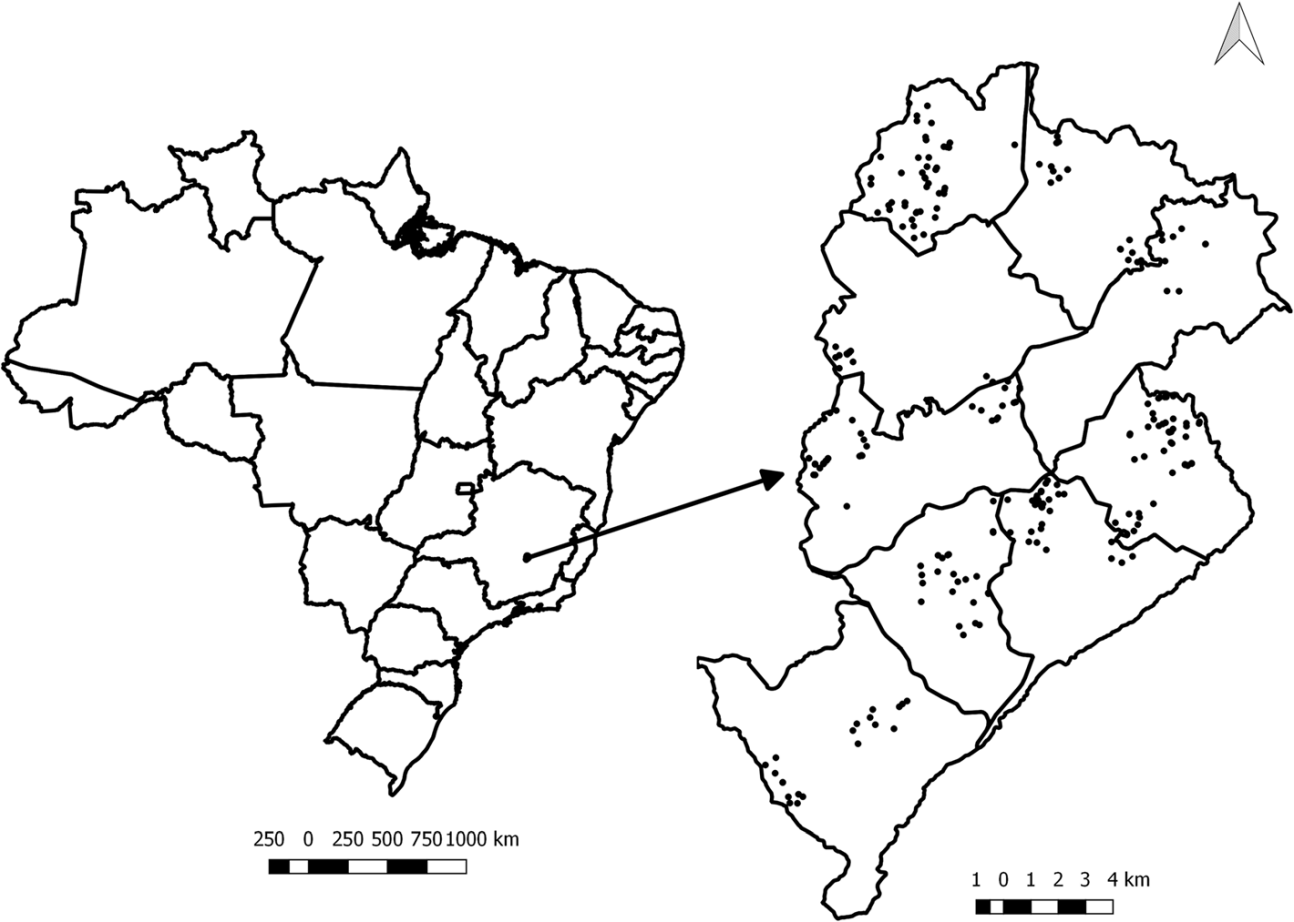
Distribution of fruits and vegetables food stores and Health Academy Program centers

To define the food environment of the 18 HAP centers, we used their geographical positions, obtained by the geographical coordinates in the Google Earth program, and created buffers with 1 mile (1600 m) around each center. Further information regarding the food environment can be found in greater detail in another publication [16]. All establishments selling fruits and vegetables (FV) within these buffers, such as large-chain supermarkets, specialized FV markets/stores or open-air food markets, local markets, convenience stores, and bakeries, were included in the study (Fig. 1).

All frequent HAP users (adults and elderlies) who had participated in the practice of physical exercise in the previous month to the beginning of data collection and residing up to 1 km from the investigated food stores were analyzed in this study. This selection aimed to include users who lived in the HAP territory, corresponding to the social context investigated. This distance was chosen as it is a reasonable walking distance [18].

### Data collection

The information used in the study was obtained via two procedures. The first was based on face-to-face interviews with HAP users, and the second involved direct observation of food stores in HAP neighborhoods. Information regarding the interview guide of the food environment can be found in greater detail in another publication [19].

Data on HAP users were obtained via a pre-coded and pre-tested protocol adapted from national surveys. Sociodemographic variables, such as age, sex, years of education, occupation, and family income, were examined. Monthly income per capita was determined by dividing the monthly income by the number of household members, expressed in dollars. Those measures were aggregated to describe HAP participants. Additionally, participants’ home addresses were collected.

From direct observation of the food environment, we found that the food stores that sold fruits and vegetables included in the study were as follows: stores registered in georeferenced databases provided by the Municipal Collection Department of the municipality; open-air food markets obtained from the City Hall website and stores that were not registered in public databases but were identified in on-site observations by field staff [16].

Data collected for the community nutrition environment included locations and types of food stores. The store types were classified as follows: a) large-chain supermarkets (commercial establishment of food in large areas of intense flow and easy access, presenting greater supply and lower prices); b) specialized FV markets/stores or open-air food markets (fixed and mobile establishment, specializing in the distribution of horticultural products); c) local markets (old and traditional establishment that supply small domestic emergencies); and d) convenience stores and bakeries (retail trade usually located in gas stations, with sales of processed foods and manufacture of bakery products) [8].

For the evaluation of access to commercial establishments, we used the Healthy Foods Index (HFSI), composed of twelve variables related to the consumer nutrition environment, concerning the availability, variety, and advertising of healthy food (such as FV) and ultra-processed products (sugary drinks, corn snacks, and cookies filled with chocolate) [8]. This index was validated for the Brazilian context. Kappa statistics for inter-rater reliability ranged from 0.66 to 0.95. Test-retest reliability was likewise high, with kappa statistics ranging from 0.61 to 1.00 [20]. HFSI scores ranged from 1 to 16, with higher values indicating better access to healthy food [8].

### Data analysis

The distance of users’ homes to the food stores was calculated using the Near command, the proximity analysis tool in the ArcView software, which creates the value of the line distance unit between two points. Running the tool turns two fields into an attribute table; one identifying the nearest point, and the other describing the calculated distance within which one can obtain a descriptive proximity analysis.

A descriptive analysis of data and variable distribution was conducted following a normality assessment using the Kolmogorov-Smirnov test. All variables showed asymmetrical distribution and were expressed for conglomerate data via median, minimum, and maximum values.

Multiple linear regression analysis was performed to examine the association between access to healthy foods, measured for HFSI index (outcome variable), the aggregate data on users’ income per capita, and the food store type (proportions of large-chain supermarkets, specialized FV markets/stores and open-air food markets and local markets). In the final model, only the variables that showed 5% statistical significance, determined via analysis of variance, remained. The quality adjustment was performed using the coefficient of determination (R^2^ adjusted). The residuals were evaluated according to the assumptions of normality, homoscedasticity, linearity, and independence. Diagnostics were performed to identify possible outliers. Scatter plots, and partial diagrams were constructed to identify possible breaches of assumption, and verification of interaction and multicollinearity was performed for the variables included in the model.

Data were tabulated using Access 2010®, georeferenced using ArcView® software (version 10.1), and analyzed using ArcView® and the Statistical Package for Social Sciences (SPSS)® program for Windows (version 19.0: SPSS, Inc. Chicago, III).

The study was approved by the Research Ethics Committee at the Universidade Federal de Minas Gerais (0537.0.0203.000–11), and City Hall in the municipality (0537.0.0203.410-11A).

## Results

The study included 2831 users in 18 centers. The majority (88.3%) were women, with a median age of 58 years (range: 21–89 years) and 8 years of education (range: 4–11 years). With respect to professional occupation, 34.6% were retired or pensioners, 35.1% were employed, and 28.6% performed unpaid activities in their homes. The median per capita income monthly was $278.90, ranging from $202.20 to $591.53 (Table 1).

**Table 1:** Sociodemographic characteristics of participants

Region	HAP*	Users (n)	Sex (%)		Age**	Income per capita ($)**	Years of Education**
			Female	Male			
A	1	23	91.3	8.7	56.0	205.68	5.0
	2	203	81.3	18.7	57.4	308.52	7.0
B	1	116	84.5	15.5	65.0	591.53	11.0
	2	108	82.4	17.6	61.0	274.23	5.0
C	1	153	85.0	15.0	59.0	329.06	9.0
	2	194	31.2	8.8	59.0	308.52	9.5
D	1	230	82.8	17.8	55.0	257.10	7.0
	2	148	89.2	10.8	55.0	246.81	6.5
E	1	271	91.5	8.5	56.0	299.74	8.0
	2	103	93.2	6.8	64.0	278.90	7.0
F	1	222	94.1	5.9	59.0	278.90	5.0
	2	40	92.5	7.5	53.5	202.20	6.0
G	1	194	91.2	8.8	62.5	370.22	8.0
	2	131	85.5	14.5	55.0	205.68	4.0
H	1	187	87.7	12.3	55.0	219.39	7.0
	2	126	91.3	8.7	58.0	370.22	8.0
I	1	205	92.7	7.3	58.0	297.82	8.0
	2	177	84.7	15.3	60.0	297.95	7.0
Total	–	2831	88.3	11.7	58.0	278.90	8.0

* Health Academy Program centers

**Median

Considering the food stores surveyed, 61.3% were specialty FV markets/stores or open-air food markets, followed by markets or large-chain supermarkets (20.5%) and local markets (17.6%) (Table 2).

**Table 2:** Food stores with fruits and vegetables in the Health Program Academy neighborhoods

Region	HAP*	Food store (n)	Type of store (%)		
			Large-chain supermarkets	Specialty fruit and vegetable markets or open-air food markets	Local Markets
A	1	10	20.0	50.0	30.0
	2	10	10.0	70.0	20.0
B	1	48	21.3	72.3	6.4
	2	12	0.0	75.0	25.0
C	1	33	21.2	63.6	15.2
	2	31	25.8	61.3	12.9
D	1	17	17.6	52.9	29.4
	2	16	12.5	50.0	37.5
E	1	18	33.3	61.1	5.6
	2	10	20.0	60.0	20.0
F	1	11	18.2	36.4	45.5
	2	09	11.1	44.4	44.4
G	1	17	23.5	76.5	0.0
	2	22	36.4	50.0	13.6
H	1	10	20.0	70.0	10.0
	2	15	35.7	35.7	28.6
I	1	31	12.9	74.2	12.9
	2	16	12.5	62.5	25.0
Total	–	336	20.5	61.3	17.6

* Health Academy Program centers

The median HFSI value was 11 (range = 5–16). Regarding the type of stores, specialized FV markets, and open-air food market showed better HFSI (13: range = 7–16), followed by large supermarket chain (8: range = 5–11) and local markets (7: range = 5–16).

Multiple linear regression analysis showed that the type of food store, represented by specialized FV markets/stores or open-air food markets (*p* < 0.001) and supermarkets (*p* < 0.05), positively influenced access to healthy food, accounting for approximately 70% of the variation in access. Therefore, an increase of 1% in specialized FV markets/stores or open-air food markets and supermarkets raised HFSI values by 0.12 and 0.07 points, respectively. Income did not influence the HFSI values of commercial establishments (Table 3).

**Table 3:** Multiple linear regression between access to healthy foods

Variable	Coefficient (β)	Standard error	Standardized beta	*p* value
Constant	1.943	1.418	–	0.191
Supermarket (%)	0.067	0.026	0.360	0.023
Specialized FV markets or open-air food markets (%)	0.123	0.019	0.909	< 0.001

note: coefficient of determination (R^2^ = 0.732; R^2^_adjusted_ = 0.697; p < 0.001); Shapiro-Wilk normality test (*p* = 0.814).

## Discussion

Access to healthy food, measured by HFSI, was positively influenced only by the type of food store, indicating that an increase in the number of specialty FV markets/stores, open-air food markets, and supermarkets could promote better access to healthy food in the areas examined.

The local food environment analyzed revealed a predominance of specialty FV markets/stores or open-air food markets within HAP neighborhoods [16]. This result might be accounted for by the public policies for food and nutritional security presented in the city, which are recognized internationally. This policy has a set of actions, including the implementation of specialty FV markets and open-air food markets in the city, which offer healthy food at lower prices [21]. The open-air food markets are weekly street markets that usually sell fresh products such as FV, meat, and fish. The specialized FV markets/stores sell an average of 70 items, with 20 horticultural products sold at a maximum price of $0.40 per kilo [22]. Nevertheless, the open-air markets were distributed unevenly across the municipality. Of the 64 existing units, 35 were located in the central area of the city, while other more remote and poorer regions (A region and F region) included only one unit. A similar distribution was found in another Brazilian ecological study that aimed to describe local food environment variables in the city of Sao Paulo [13]. Of these 64 units, 13 were located in HAP areas, and one was no longer in operation (I region). The results also showed that some characteristics of the open-air markets surveyed were unfavorable, as they were small, with an average of four tents (range: 2–9); sold only one product, such as fruit, vegetables, meat, fish, or biscuits; and sold ultra-processed food, such as biscuits, cookies and sweets.

The fact that the open-air markets sell only one product (fruits, vegetables, meat, fish) and ultra-processed food, coupled with their lower distribution within the remote and poor areas, demonstrates the need for a policy review to minimize the inequalities of material and social resources and to promote changes in the territory characteristics. An increased number and a higher quality of stores in areas with poor accessibility would promote access to fresh products, such as FV, in populations with low SES [6, 8].

Health promotion action carried out in the HAP, such as activities to promote healthy eating and community education, might present unsatisfactory results when performed in environments that do not provide healthy options [16]. Changes in eating habits can be difficult to achieve, as they depend on both the availability and quality of food in the neighborhood [23, 24]. Some cross-sectional studies, conducted with a population from the Danville River district and with the participants of the Atherosclerosis Risk in Communities Study, have suggested an association between the presence of food stores that offer healthier food and lower prevalence of overweight and obesity [25, 26]. Moreover, the implementation of these stores could improve the local economy, provide jobs for residents, increase the local base tax, attract other forms of retail, offer greater availability of food at lower prices, and increase residents’ purchasing power [12].

The influence of socioeconomic data on the HFSI of HAP users was not observed, possibly due to the similarity between the participants of the HAP with respect to per capita income and schooling. Similarly, a cross-sectional study conducted in Danville (USA) did not find differences in the availability of commercial establishments with socioeconomic data, but noted that there might be distinctions regarding the variability, quality, and price of the available products [27], which reinforces the importance of understanding primary aspects of the food environment, such as quality, availability, and variety of food for the proper understanding and monitoring of the food environment.

One limitation of this study was that the geographical locations of some HAP centers were close to other municipalities without georeferenced data, which might have led to an underestimation of the number of food stores. Also, we cannot guarantee that all non-registered stores in the county prefecture database were analyzed. However, we conducted an assessment of the spatial distribution of these stores to verify if they were randomly distributed throughout the buffer, which indicated collection throughout the neighborhood.

The use of a buffer in determining the food environment implies determined and recognized boundaries that cannot be restricted to these limits. Thus, the area of coverage and, therefore, access can be smaller or larger depending on the arbitrary determination of this threshold. We did not investigate the establishments in which participants purchase food. We attempted to collect this information, but the users could not reference the shopping store. However, previous qualitative research conducted in these neighborhoods revealed individuals purchased FV from shops close to their houses [28]. Also, it is important to consider that for the buffer; we used a similar radius value as that which is used in the literature [29, 30]. A radius of 1 km from the residence of the HAP users was used, since this is a reasonable distance to travel by foot [18].

Another limitation refers to the use of solely socioeconomic data from HAP users to determine the neighborhood characteristics, which might be different from non-HAP users. The use of socioeconomic data from HAP users created a sample of low socio-economic variability. However, we believe this choice was more robust: the use of primary data was important to describe the unstudied vulnerable areas.

Ecological studies are fundamental in linking the environment with access to healthy food, such as FV, particularly in Latin American countries, in which such studies are scarce [9]. It was also considered essential to collect data via direct observation, which allowed an understanding of real access and the quality of the consumer nutrition environment [31]. Examination of other aspects of the food environment, such as the quality, availability, and variety of food, can contribute to the understanding and monitoring of food environments.

## Conclusion

Access to healthy food in food stores (HFSI index measured in the consumer nutrition environment level) appeared to be associated only with the type of stores involved. These results highlight the important influence exerted by specialty FV markets/stores or open-air food markets and supermarkets in the construction of a healthy eating environment, particularly in areas with poor access to healthy food. We highlighted the importance of public policies related to food supply, which prioritize areas with poor access and aim to improve access, availability, and consumption of healthy food, such as FV, contributing to overcoming inequality in access.

## Abbreviations

FV: Fruits and vegetables
HAP: Health Academy Program
HFSI: Healthy Foods Index
SES: Socioeconomic status
